# A case of pulmonary histoplasmosis diagnosed after lung lobectomy

**DOI:** 10.1186/s40792-018-0554-9

**Published:** 2018-12-20

**Authors:** Shun Tanaka, Ryo Kobayashi, Hiroyuki Nagita, Kazuaki Okamoto, Yuki Iida, Kumiko Hongo, Yukio Ishihara, Yusuke Kita, Naoki Takabayashi, Ken Kuriki, Takeyui Hiramatsu

**Affiliations:** 1 Department of Surgery, Yaizu City Hospital, 1000, Dobara, Yaizu, Shizuoka Japan; 2 Department of Pathology, Yaizu City Hospital, 1000, Dobara, Yaizu, Shizuoka Japan

**Keywords:** Pulmonary histoplasmosis, *Histoplasma capsulatum*, Imported mycoses, Lung cancer, Metastatic lesions

## Abstract

**Background:**

Histoplasmosis is considered a fairly rare imported mycosis in Japan. Here we report a case of histoplasmosis describing the preoperative findings, histopathological findings, supposed infection route, and appropriate treatment, including the postoperative management.

**Case presentation:**

A healthy 65-year-old man was found at routine medical check-up to have an abnormal opacity on chest radiography. A chest computed tomography (CT) scan showed a nodular lesion in the posterior basal segment of the right lung, as well as two smaller nodules in the same lobe. This was highly suggestive of primary lung cancer with pulmonary metastases in the same lobe. We thus performed a right lower lobectomy with hilar and mediastinal lymph node dissection via thoracotomy. The lesions were diagnosed as pulmonary histoplasmosis on histopathology. At 6-month follow-up examination, the patient was free from fungal infection without any postoperative medication.

**Conclusions:**

We describe a patient with pulmonary histoplasmosis diagnosed following surgical lobectomy. The possibility of pulmonary histoplasmosis should be considered in the differential diagnosis of pulmonary nodular lesions.

## Background

Histoplasmosis is a fungal infection caused by *Histoplasma capsulatum* (*H. capsulatum*). *H. capsulatum* is a soil-based fungus that has been isolated from many regions of the world and is most often associated with river valleys; the most highly endemic regions are the Ohio and Mississippi River Valleys [1]. In Japan, histoplasmosis is classified as an imported infectious disease, and the number of patients with histoplasmosis has increased dramatically since the mid-1980s, with a total of 83 cases reported as of August 2015 [2].

## Case presentation

**Fig. 1:**
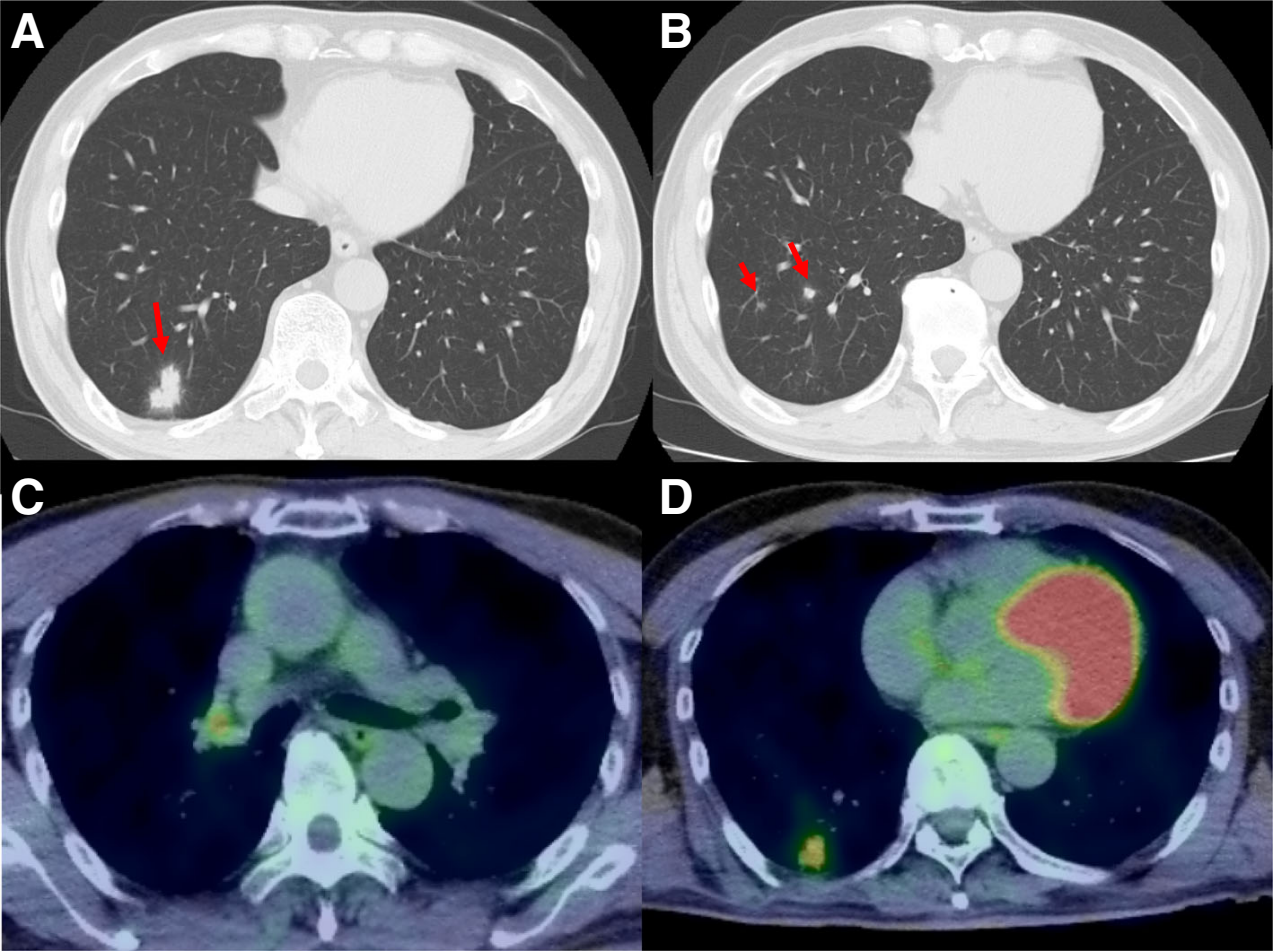
Imaging findings. **a** Computed tomography showing the pulmonary nodule (24 mm) in the posterior basal segment of the right lung. **b** Computed tomography showing two small nodules (8 mm and 6 mm) in the posterior basal segment of the right lung. **c** Fluorodeoxyglucose positron emission tomography showing abnormal uptake in the main nodule. **d** Fluorodeoxyglucose positron emission tomography showing abnormal uptake in right hilar lymph nodes

A 65-year-old man with no significant past medical history underwent chest radiography at routine medical check-up. This revealed a nodular opacity in the right lung field. He was referred to our department for further examination. He had presented no symptoms such as fever, dyspnea, dysphagia, weight loss, or hemoptysis. He worked in a construction company and had travelled to Taiwan 2 years previously. He had two cats as pets. He had smoked one pack of cigarettes per day for 20 years. His physical findings, tumor markers, and other laboratory tests were unremarkable. Spirometry test showed normal pulmonary function. The first computed tomography (CT) scan showed a nodule of 24 mm in diameter with an irregular and spiculated border in the posterior basal segment of the right lung (Fig. 1a), and two smaller nodules (8 mm and 6 mm) in the same lobe (Fig. 1b). One month later, the main tumor had enlarged to 27 mm in size, and the others to 10 and 7 mm. The head magnetic resonance imaging (MRI) showed no intracranial mass. The fluorodeoxyglucose positron emission tomography (FDG-PET) showed abnormal uptake in the main nodule (24 mm) (Fig. 1c) and the right hilar lymph nodes (Fig. 1d).

Thus, a diagnosis of primary lung cancer with intralobar metastases and ipsilateral hilar lymph node metastases was made, and he underwent right lower lobectomy with hilar and mediastinal lymph node dissection via thoracotomy. We inserted a chest drainage tube intraoperatively. Ampicillin/sulbactam was administered only on the day of surgery as prophylactic treatment.

At thoracotomy, a hard mass adjacent to the pleura was observed in pulmonary segment 10, but no other specific abnormalities were found.

**Fig. 2:**
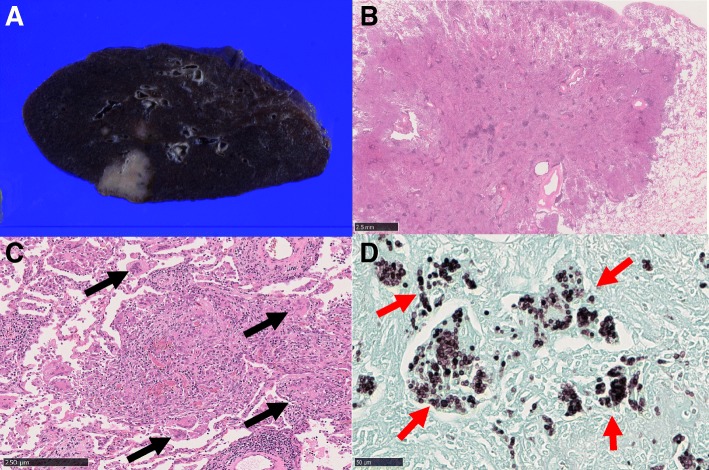
Pathological findings. **a** The 24 × 22 × 20 mm mass adjacent to the pleura. **b** Hematoxylin and eosin (HE) stain × 0.75. The epithelioid granuloma which include vessels and bronchi. **c** HE stain × 20. There are multinucleated giant cells in the granuloma (black arrows). **d** Grocott’s methenamine silver stain × 40. There are a lot of yeast-like fungi (red arrows) in the cytoplasm of multinucleated giant cells and foam cells

Histopathological analysis revealed well-circumscribed nodular lesions with noncaseating epithelioid cell granulomas, without features of malignancy. There were numerous small yeast-like fungi stained by Grocott’s methenamine silver procedure in the granulomas. Alcian blue and the Mucicarmine dye failed to show capsules around them. These findings and the forms of the fungi led to the diagnosis of lung histoplasmosis (Fig. 2). No fungi were detected in the excised lymph nodes.

The patient was discharged on postoperative day 20. Length of hospital stay was prolonged due to a persistent pleural effusion. The amount of pleural effusion drainage was 410 ml on postoperative day 1. More than 200 ml per day had been drained until on postoperative day 13. The pleural effusion was clear and pale yellow. We removed the chest tube on postoperative day 16. No antifungal drugs were administered after surgery. At 6-month follow-up, he did not show any signs of relapse.

## Discussion

This is a case report describing histoplasmosis in a Japanese male who presented with one large and two smaller lung nodules mimicking lung cancer and intralobar metastases.

The route of infection in this patient is unclear. He had travelled to Taiwan 2 years prior to presentation. However, reported cases of histoplasmosis in Taiwan are very rare, with only seven cases as of 2006 [3]. Furthermore, the patient does not recall any symptoms after returning from Taiwan, making it unlikely that he contracted the infection there. We considered the possibility of his contracting the infection from his pet cats. However, feline histoplasmosis is very rare in Japan, with only one case reported to date [4]. Moreover, his cats did not present any symptoms suggestive of histoplasmosis. He worked in a construction company, so he might have inhaled the fungus body from contaminated soil. However, there are very few reported cases of domestic infection, who had no overseas travel history, only five as of 2008 [5]. Ultimately, we are unable to determine the route of infection definitively—he had not travelled to endemic regions, and other considered sources of the infection would be very rare.

The radiographic findings of pulmonary histoplasmosis are not inconsistent with those of metastatic lesions. Croft et al. reported that FDG-PET is not useful in distinguishing histoplasmosis from lung cancer in regions of high prevalence [6]. In our case, we expedited surgery, firstly because there was a high index of suspicion of lung cancer from imaging studies and secondly because the nodules were increasing rapidly in size. In retrospect, there might have been an option to perform intraoperative rapid pathological examination to confirm or exclude the possibility of malignancy.

The vast majority of cases of acute pulmonary histoplasmosis do not require therapeutic intervention except for individuals whose immune systems are compromised. Oral itraconazole is administered to those who do not recover 1 month after the onset of the disease, or who exhibit hypoxemia [7]. As we had considered that the patient had lung cancer rather than pulmonary fungal disease prior to surgery, we administered prophylactic antibiotic treatment (ampicillin/sulbactam) as prevention of surgical site infection.

*H. capsulatum* is known to spread through lymphatic pathways. However, the hilar lymph nodes were not involved with fungi in our case, although abnormal uptake was seen on FDG-PET.

The length of hospital stay was prolonged due to persistent pleural effusion. Repeated cultures of the pleural effusion were all negative, and the patient had no symptoms of pyothorax, fever, or chest pain. The patient did not have heart failure or chylothorax too from physical findings or the color of the pleural effusion. We consider the etiology of pleural effusion might be lymphorrhea following lymph node dissection. The size of pleural effusion might decrease spontaneously as healing of wound or adhesion of the lung to mediastinum progressed. The other possible etiology is infection. Resection of the infectious tissue might cause local pleurisy, which lead to pleural effusion. The culture of pleural effusion could be false-negative due to the low culture positive rate of histoplasmosis.

There are no clear-cut criteria as to whether postoperative antifungal drugs are necessary or not. We did not administer postoperative antifungal drugs because the patient was not immunocompromised, and because the infected lesion was histopathologically confined to the resected lung lobe. There were no signs of relapse at 6-month follow-up.

In conclusion, we report a case of a healthy Japanese male diagnosed with pulmonary histoplasmosis after lung lobectomy. Histoplasmosis itself occurs rarely in Japan, and in addition, imaging studies cannot distinguish its pulmonary manifestations from those of lung cancer. We need to bear in mind the possibility of histoplasmosis in cases of pulmonary nodular lesions.

## Abbreviations

CT: Computed tomography
FDG-PET: Fluorodeoxyglucose positron emission tomography
*H. capsulatum*: *Histoplasma capsulatum*
MRI: Magnetic resonance imaging

## References

[1] Ajello L, Ajello L, Chick W, Furculow MF (1971). Distribution of Histoplasma capsulatum in the United States. Histoplasmosis.

[2] The trend of imported mycoses in Japan. Medical Mycology Research Center, Chiba University. http://www.pf.chiba-u.ac.jp/clinical/mycosis.html (in Japanese). Accessed 30 May 2017.

[3] Lai C-H, Lin H-H (2006). Cases of histoplasmosis reported in Taiwan. J Formos Med Assoc.

[4] Kobayashi R, Tanaka F, Asai A (2009). First case report of histoplasmosis in a cat in Japan. J Vet Med Sci.

[5] Nishikawa T, Muramatsu T, Matsumi A, Inoue F (2009). A case of pulmonary histoplasmosis difficult to differentiate with lung cancer. JJACS.

[6] Croft DR, Trapp J, Kernstine K (2002). FDG-PET imaging and the diagnosis of non-small cell lung cancer in a region of high histoplasmosis prevalence. Lung Cancer.

[7] Wheat LJ, Freifeld AG, Kleiman MB (2007). Clinical practice guidelines for the management of patients with histoplasmosis: 2007 update by the Infectious Diseases Society of America. Clin Infect Dis.

